# Editorial: Women in science—Gastroenterology 2022

**DOI:** 10.3389/fmed.2022.1013494

**Published:** 2022-10-03

**Authors:** Stefania Chetcuti Zammit

**Affiliations:** Gastroenterology Department, Mater Dei Hospital, Imsida, Malta

**Keywords:** gastroenterology, women, cirrhosis, inflammatory bowel disease, colorectal cancer

This Editorial encompasses highlights from a Research Topic of articles published in *Women in science—Gastroenterology 2022*. The main aim of this Research Topic of Frontiers in Medicine is to promote high quality research by women in the field of gastroenterology. Articles were selected based on the relevance of the Research Topic to the current practices in gastroenterology and female authorship. Over the years, females have been instrumental in advancements in the field of gastroenterology including the improvement in endoscopic procedures, the investigative approach of patients with underlying gastrointestinal pathology and drugs used in the management of such patients.

Females who choose to pursue a career in gastroenterology are faced with challenges, at times struggling to publish and to progress further in their career. Here we celebrate the inspiration of these authors, their resilience and dedication, qualities that have allowed them to be successful and to meet the competitive demands of their workplaces. We hope that their example will serve as a role model for younger male and female gastroenterologists alike in their everyday clinical practice.

*Role of galectins in the liver diseases: A systematic review and meta-analysis* (An et al.)—Galectins are responsible for the regulation of pre-messenger RNA (mRNA) splicing, cell cycle, cell growth, and cell apoptosis and the development and/or progression of cancer. Carbohydrate recognition domains in galectins play a regulatory role in liver diseases by binding to glycoconjugates expressed in hepatocytes. This met-analysis explores the role of galectins in liver related disorders, namely in predicting prognosis in hepatocellular carcinoma that has a poor 5 years survival. Identifying these biomarkers provides a stepping stone for potential therapeutic targets to manage chronic liver disorders and hepatocellular carcinoma.

*Genetic polymorphisms and clinical features in diabetic patients with fatty liver: Results from a single-center experience in Southern Italy* (Villani et al.)—Non-alcoholic fatty liver disease (NAFLD) has a high prevalence in patients with diabetes and the metabolic syndrome. It is a major burden in the medical field in view of the ongoing monitoring that this condition entails and the potential requirement for liver transplantation. This single study highlights the role of genetic studies in the early identification of patients at a higher risk of cirrhosis and a lower estimated glomerular filtration rate who will require closer surveillance.

*Exposure to plasma from non-alcoholic fatty liver disease patients affects hepatocyte viability, generates mitochondrial dysfunction, and modulates pathways involved in fat accumulation and inflammation* (Grossini et al.)—In this study, the authors determine whether factors in the plasma of NAFLD patients may induce a similar phenotype in hepatocytes by activation of intracellular inflammatory pathways. Although further studies are required, this mechanism can influence cell viability and results in activation of pathways causing inflammation and tissue damage.

*Risk factors of invasive fungal infection in recipients after liver transplantation: A systematic review and meta-analysis* (Liu et al.)—An improvement in surgical technique has resulted in an improved outcome following liver transplantation. However, fungal infection remains a major cause of morbidity and mortality in this cohort of patients. Results from existent studies are limited by geographical limitations and sample sizes. In this meta-analysis, the authors analyzed existent studies to identify risk factors for fungal infection following liver transplantation, thus providing a basis for clinical prevention.

*Combined estrogen alpha and beta receptor expression has a prognostic significance for colorectal cancer patients* (Topi et al.)—This study investigated the prognostic significance of the combined expression of estrogen receptor alpha (ERα) and estrogen receptor beta (ERβ) in female patients with colorectal cancer. The authors investigated survival, local recurrence and liver metastasis in relation to the estrogen receptor expression. This study provides a basis for future research on the early detection of colorectal cancer, targeted therapies and prognostic markers to predict the recurrence risk more accurately.

*An assessment of physicians' recommendations for colorectal cancer screening and international guidelines awareness and adherence: results from a Thai national survey* (Pausawasdi et al.)—Screening for colorectal cancer (CRC) is a proven strategy to improve prognosis and survival as lesions can be detected in a precancerous phase and there is the ability to cure precancerous lesions mostly by endoscopically. The uptake of CRC screening is generally low in the Asia Pacific region. The aim of this study was to understand the recommendations by physicians in Thailand for CRC screening and the awareness and adherence to international guidelines by means of a questionnaire. The authors tried to understand how recommendations vary across different specialities and if low uptake of CRC screening could potentially be improved by understanding trends in recommendations by physicians.

*Circulating fibroblast activation protein as potential biomarker in patients with inflammatory bowel disease* (Corsi et al.)—This study explores the role of circulating Fibroblast activation protein (FAP), a serum biomarker that correlates inversely with disease activity in inflammatory bowel disease (IBD). It can serve an important role as a non-invasive test in those with suspected IBD and in predicting recurrence following surgery. Unlike other biomarkers such as faecal calprotectin, it is significantly specific to IBD, distinguishing IBD related inflammation from other causes of colitis. Serum biomarkers are increasingly sought after in current medical practice as they minimize the requirement for invasive procedures and their associated complications, and improve cost effectiveness.

*Are steroids still useful in immunosuppressed patients with inflammatory bowel disease? A retrospective, population-based study* (Sicilia et al.)—Patients with IBD who are on immunosuppressants may require systemic steroids to manage moderate to severe flare ups. The aim of this study was to assess the effectiveness of steroids in this scenario and to determine whether the use of steroids can impact on the requirement for further escalation of therapy (the requirement for biologics). The authors question whether such treatment that has been used for a long time in the management of patients with IBD still plays an important role in managing flare ups and minimizes the need for escalation of therapy.

*Helicobacter pylori eradication therapy affect the gut microbiota and ghrelin levels* (Martín-Núñez et al.)—In this prospective study, the authors analyzed the effect of *Helicobacter pylori* eradication eherapy on ghrelin levels that correlate with the changes in diversity and abundance of gut microbiota. Ghrelin can affect stimulation of food intake, growth hormone secretion, adiposity, gastric motility, acid secretion and insulin secretion inhibition. Studying the association between ghrelin levels and gut microbiota can shed light on the pathogenesis of obesity, varying gut microbiota and ghrelin levels.

## Author contributions

The author confirms being the sole contributor of this work and has approved it for publication.

## Conflict of interest

The author declares that the research was conducted in the absence of any commercial or financial relationships that could be construed as a potential conflict of interest.

## Publisher's note

All claims expressed in this article are solely those of the authors and do not necessarily represent those of their affiliated organizations, or those of the publisher, the editors and the reviewers. Any product that may be evaluated in this article, or claim that may be made by its manufacturer, is not guaranteed or endorsed by the publisher.

